# Gene Silencing of Porcine *MUC13* and *ITGB5*: Candidate Genes towards *Escherichia coli* F4ac Adhesion

**DOI:** 10.1371/journal.pone.0070303

**Published:** 2013-07-29

**Authors:** Chuanli Zhou, Zhengzhu Liu, Yang Liu, Weixuan Fu, Xiangdong Ding, Jianfeng Liu, Ying Yu, Qin Zhang

**Affiliations:** 1 Key Laboratory of Animal Genetics, Breeding and Reproduction, Ministry of Agriculture, National Engineering Laboratory for Animal Breeding, College of Animal Science and Technology, China Agricultural University, Beijing, P.R. China; 2 Department of Animal Science, College of Animal Science and Technology, Hebei Normal University of Science and Technology, Changli, P.R. China; Thomas Jefferson University, United States of America

## Abstract

**Background:**

Integrin beta-5 (*ITGB5*) and mucin 13 (*MUC13*) genes are highly expressed on the apical surface of intestinal epithelia and are thought to be candidate genes for controlling the expression of the receptor for enterotoxigenic *Escherichia coli* (ETEC) F4ac. Human MUC13 protein has an expected role in protecting intestinal mucosal surfaces and porcine ITGB5 is a newly identified potential receptor for ETEC F4ac.

**Methodology/Principal Findings:**

To test the hypothesis that *ITGB5* and *MUC13* both play key roles in protection of the intestinal mucosa against pathogenic bacterium, porcine intestinal epithelial cells (IPEC-J2) were transfected with *ITGB5*-targeting, *MUC13*-targeting or negative control small interfering RNA (siRNA), respectively. Firstly, we measured mRNA expression levels of mucin genes (*MUC4*, *MUC20*), pro-inflammatory genes (*IL8*, *IL1A*, *IL6*, *CXCL2*), anti-inflammatory mediator *SLPI*, and *PLAU* after RNAi treatments with and without ETEC infection. Secondly, we compared the adhesions of ETEC to the pre- and post-knockdown IPEC-J2 cells of *ITGB5* and *MUC13*, respectively. We found that *ITGB5* and *MUC13* knockdown both had small but significant effects in attenuating the inflammation induced by ETEC infection, and both increased bacterial adhesion in response to F4ac ETEC exposure.

**Conclusions/Significance:**

Our current study first reported that *ITGB5* and *MUC13* are important adhesion molecules of mucosal epithelial signaling in response to *Escherichia coli* in pigs. These data suggest that both *ITGB5* and *MUC13* play key roles in defending the attachment and adhesion of ETEC to porcine jejunal cells and in maintaining epithelial barrier and immunity function.

## Introduction

Enterotoxigenic *Escherichia coli* (ETEC) is recognized as a common cause of diarrhoea in humans and swine, their adherence to intestinal cells is mediated by proteinaceous, species-specific colonization factors [1]. F4 fimbriae are the major colonization factors associated with porcine neonatal and postweaning diarrhoea caused by ETEC [2], [3]. Among the three different antigenic variants (F4ab, F4ac, and F4ad) of F4 fimbriae, F4ac is the most prevalent [4], [5]. ETEC having F4ac fimbriae inducing severe diarrhoea is dependent on the presence of the specific receptors for F4ac (F4acR) [6], which is encoded by the responsible gene [7]. To date, the candidate genes of F4acR having been investigated in several specific pig populations by association study between genetic markers with *in vitro* F4ac adhesion phenotypes include: mucin 4 (*MUC4*) [8], *MUC13* [7], [9], *MUC20* [10] and integrin beta-5 (*ITGB5*) [11].

Besides action as a physical barrier limiting access of microbes and toxins to the underlying tissues, the contiguous lining intestinal mucosal epithelial cells secrete long filamentous cell-surface mucins which are a major constituent of the mucus barrier [12], [13]. For these membrane anchored cell-surface mucins, in addition to protection, they play an important role in the survival of mucosal epithelial cells, and participate in intracellular signal transduction [12]. Mucin 13 is a transmembrane mucin glycoprotein highly expressed in the jejunum of pig [9]. More recently, the mRNA expression level of *MUC13* is shown to be down-regulated in the inflamed intestine cells of porcine induced by F4ac ETEC strain infection [5]. In human, MUC13 was reported to regulate epithelial inflammation in response to inflammatory and infectious stimuli [12], [13]. Being adjacent to the *MUC13* gene on porcine chromosome 13 (SSC13), *ITGB5* was our newly identified candidate gene for F4acR *via* genome wide association study [11]. As one member of the “focal adhesion” family, *ITGB5* is an attractive candidate to be tested as biomarker and/or new drug target in human pancreatic cancer [14]. In the mouse, the ITGB5 subunit was found to be expressed on both the apical and basal surface of endometrial epithelium [11], [15].

In this study, we aimed to detect the effects of deficiency of *MUC13* or *ITGB5* in porcine intestinal cells upon F4ac ETEC infection and their mucosal immunogenicity.

## Results

### Silencing of the Target Genes

**Figure 1:**
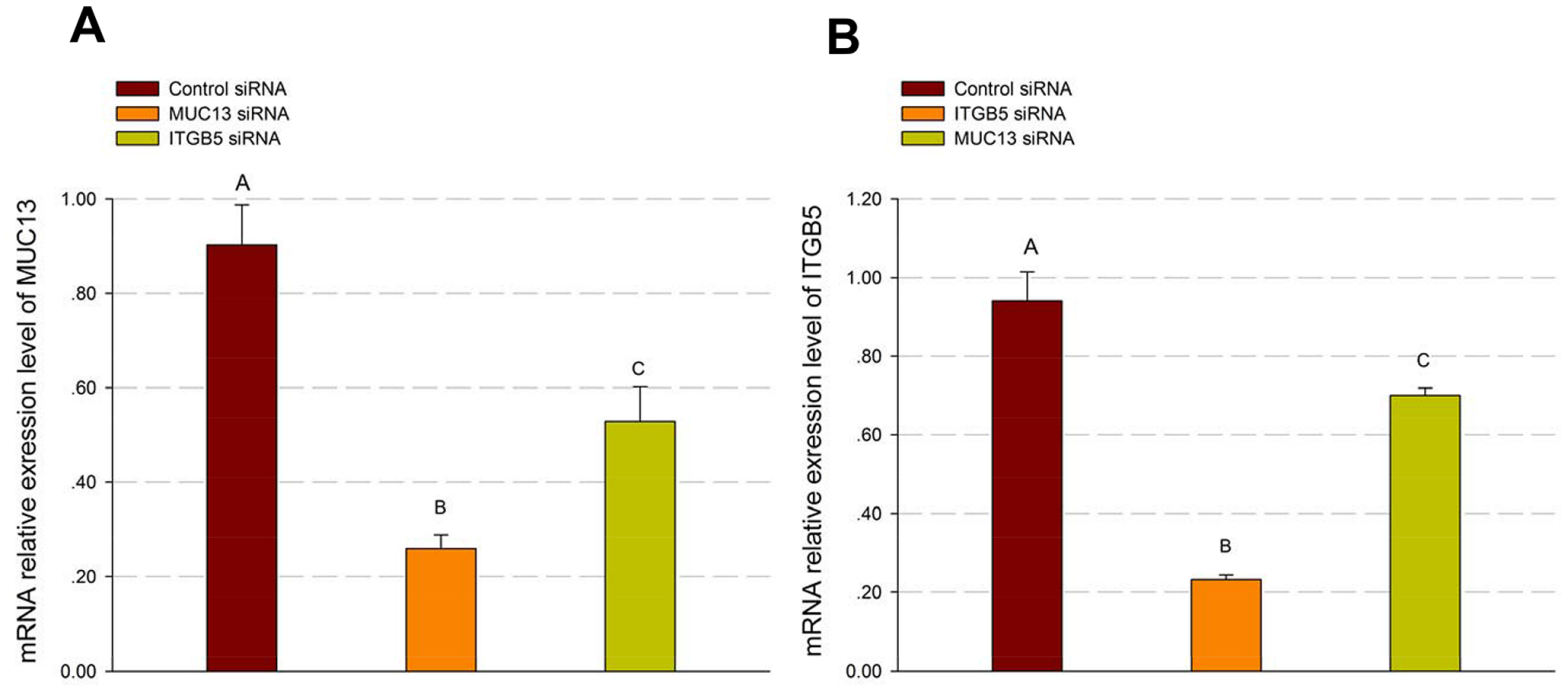
*MUC13*-targeting and *ITGB5*-targeting siRNA reduced the expression of *MUC13* and *ITGB5* in IPEC-J2 cells. IPEC-J2 cells were transfected with *MUC13*-targeting, *ITGB5*-targeting or negative control siRNA for 44 h, and the expression levels of *MUC13* (A) and *ITGB5* (B) were measured by real-time RT-PCR, respectively. For (A and B), results are the mean ± SE (standard error) from IPEC-J2 cells of three independent replicates (n = 3). In each group, values without a common letter are significantly different (*P*<0.01).

RNA interference was used to investigate the function of *MUC13* and *ITGB5* in porcine intestinal cells. Firstly, we designed and synthesized siRNAs specifically targeting porcine *MUC13* and *ITGB5*, respectively (Table 1). Subsequently, the siRNAs were transfected into IPEC-J2 cells by using Lipofectamine® RNAiMAX Reagent. In Figure 1A, we show the mRNA expression levels of *MUC13* in cells transfected with negative siRNA duplexes (negative control) and with *MUC13*-targeting siRNA, respectively, at 44 h post-transfection. The results indicated that mRNA expression of *MUC13* was significantly suppressed by the *MUC13*-targeting siRNA (Fold-change = −3.48, *P*<0.001). Compared with negative control, at 44 h post-transfection, the mRNA expression of *ITGB5* was significantly attenuated by the *ITGB5*-targeting siRNA (Fold-change = −4.05, *P*<0.001; Figure 1B).

**Table 1:** Small RNA sequences.

Target genes	GenBank Accession Number	Type	Sequence	Quantity
*MUC13*	NM_001105293.1	RNA	CCAGCUUGUUGAGGUAGAAGUAGUA	Stealth
		RNA	UACUACUUCUACCUCAACAAGCUGG	Stealth
*ITGB5*	NM_001246669.1	RNA	AAUCCGUGCAUUGGCUACAAGUUAU	Stealth
		RNA	AUAACUUGUAGCCAAUGCACGGAUU	Stealth

Because *MUC13* and *ITGB5* are located next to each other in porcine genome and can be expressed in the same cell, to examine whether there are correlation [16]/compensation [13] between the expression levels of the two adjacent genes in IPEC-J2 cells, we measured *MUC13* mRNA expression in *ITGB5* knockdown (*ITGB5*-KD) cells as well as the mRNA expression of *ITGB5* in *MUC13* knockdown (*MUC13*-KD) cells. There was a small significant decrease in the expression of *MUC13* in *ITGB5*-KD cells (Fold-change = −1.71, *P*<0.005; Figure 1A), and in the expression of *ITGB5* in *MUC13*-KD cells (Fold-change = −1.34, *P*<0.01; Figure 1B), respectively. These results demonstrated that there are potential correlation between the expressions of *ITGB5* and *MUC13* in IPEC-J2 cells.

When *MUC13* or *ITGB5* was silenced, the mRNA expression of the two genes was measured in silenced cells infected with F4ac ETEC. We observed that silence of *MUC13* decreased the expression of *MUC13* and *ITGB5*, while silence of *ITGB5* only decreased the expression of itself in the infected cells (Figure S1).

### *ITGB5* and *MUC13* Similarly Regulate Cell-surface Mucin Expression in Response to ETEC

**Figure 2:**
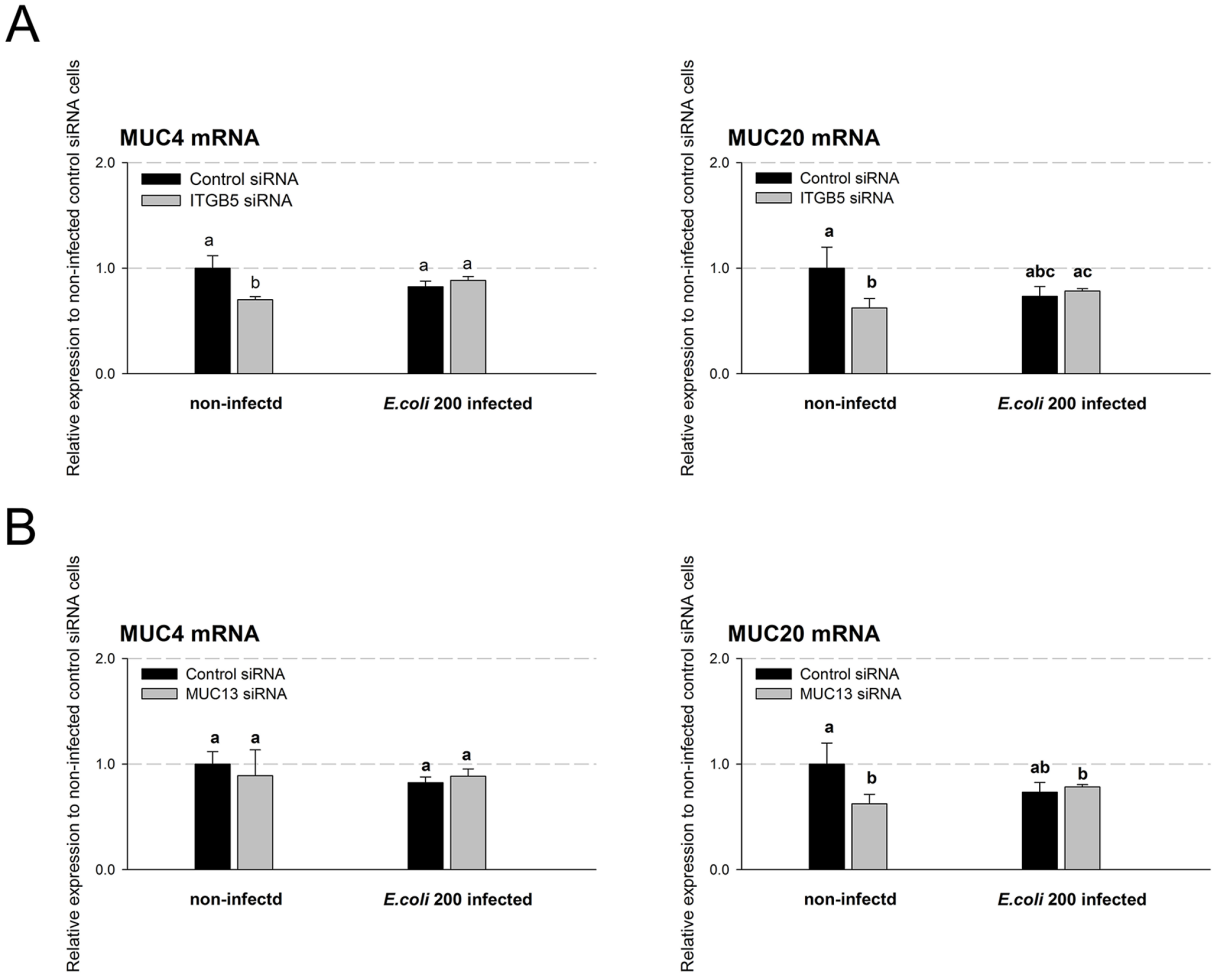
Expression of cell surface mucin genes in control and *ITGB5*-silencing/*MUC13*-silencing IPEC-J2 cells infected or non-infected with F4ac ETEC. (A) IPEC-J2 cells were transfected with *ITGB5-*targetingor negative control siRNA for 44 h and infected with or without F4ac ETEC for 3 h. Total RNA from non-infected or infected cells were used to measure the mucin genes mRNA expression by real-time PCR. (B) IPEC-J2 cells were transfected with *MUC13-*targeting or negative control siRNA for 44 h and infected and assessed as in A. Statistics: mean ± SE; n = 3; In each group, values without a common letter are significantly different (*P*<0.05).

In addition to *MUC13*, two other genes, encoding structurally similar cell surface mucins and also located on Chr13, are known to be expressed in porcine jejunum: *MUC4* and *MUC20* [3]. To identify whether there is interaction between *MUC13* or *ITGB5* with each of the two mucin genes, we firstly separately compared the mRNA expression levels of *MUC4* and *MUC20* in *ITGB5*- or *MUC13*-deficient cells with that in the negative control cells at 44 h post-transfection of siRNAs. There were small significant decreases in the expression of *MUC4* (∼30%, *P*<0.05) and of *MUC20* (∼38%, *P*<0.05) in *ITGB5*-deficient cells (Figure 2A). When *MUC13* was knocked down there was a ∼37% significant decrease (*P*<0.05) in *MUC20* mRNA expression (Figure 2B), but no significant change in *MUC4* expression. These results indicated that the expressions of *MUC4* and *MUC20* might be correlated with *ITGB5* expression and that *MUC20* might be also related to *MUC13* in IPEC-J2 cells.

Secondly, we assessed the relative effects of *ITGB5* and *MUC13* on mRNA expression of the two mucin genes (*MUC4* and *MUC20*) in response to 3 h infection with ETEC strain 200 (MOI = 8∶1) at 44 h after siRNAs transfection. After exposure to ETEC strain 200 for 3 h, no marked differences were observed in the mRNA expressions of *MUC4* and *MUC20* between negative control and *ITGB5*-deficient/*MUC13*-deficient cells, respectively.

### *ITGB5* and *MUC13* Similarly Regulate mRNA Expression of Markers of Inflammation in Response to ETEC Infection

**Figure 3:**
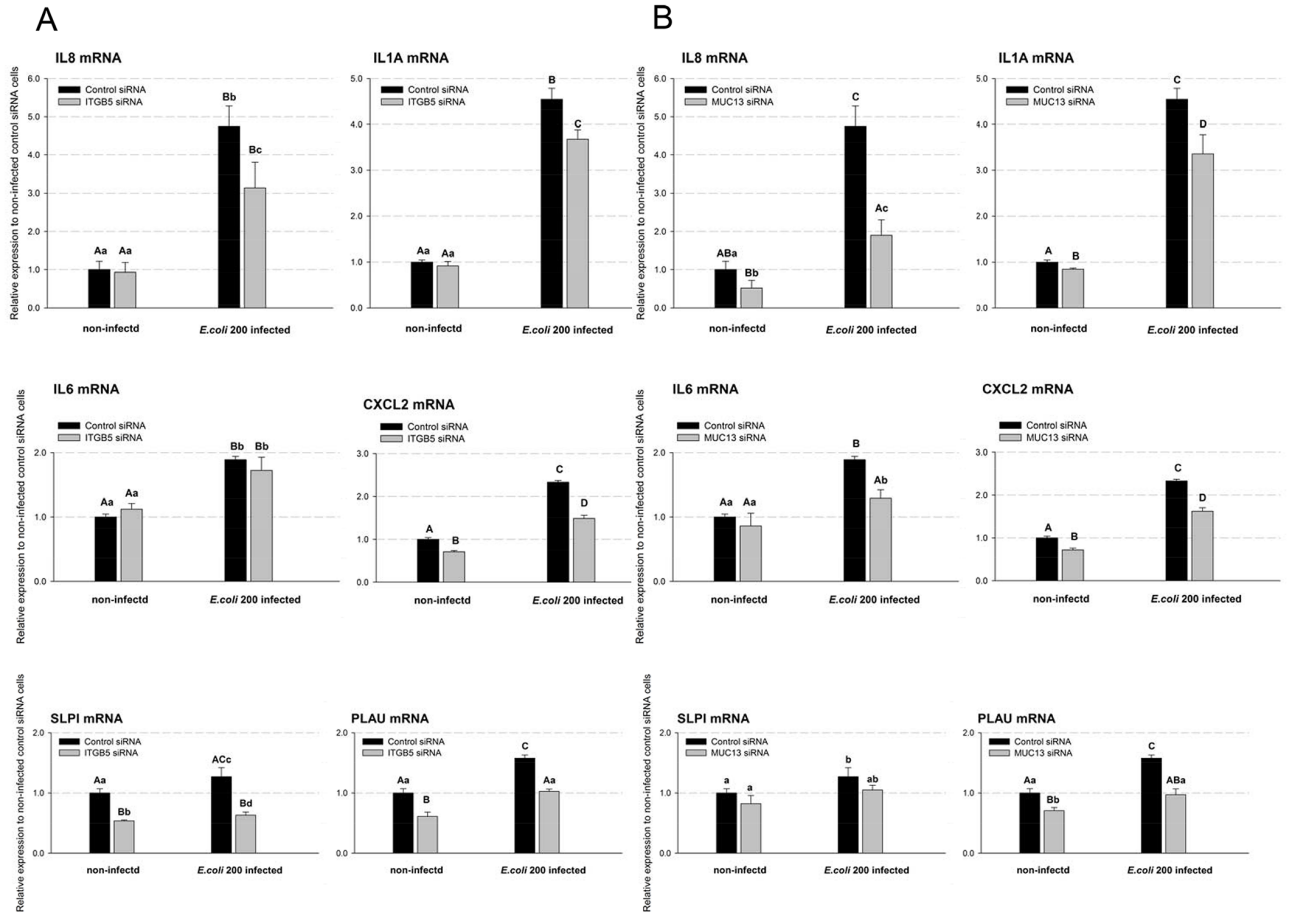
*ITGB5* and *MUC13* modulated intestinal epithelial cell inflammatory reaction in response to F4ac ETEC. IPEC-J2 cells were transfected with *ITGB5*-targeting and *MUC13*-targeting individually or negative control siRNA for 44 h and co-cultured with F4ac ETEC for 3 h or not. The mRNA levels of the inflammatory markers were measured by real-time PCR. Statistics: mean ± SE; n = 3; In each group, values without a common capital letter are significantly different (*P*<0.01), without a common lower case letter are significantly different (*P*<0.05).

To detect the effects of *ITGB5* and *MUC13* on the expressions of pro-inflammatory cytokines/chemokines, the mRNA levels of *IL8*, *IL1A*, *IL6* and *CXCL2* were measured in F4ac ETEC infected and non-infected *ITGB5*-KD or *MUC13*-KD cells, respectively. In the non-infected cells, knockdown of *ITGB5* only slightly reduced *CXCL2* mRNA expression (∼29%, *P*<0.01; Figure 3A). As a comparison, knockdown of *MUC13* significantly reduced the mRNA expression levels of *IL8*, *IL1A* and *CXCL2* (*P*<0.01; Figure 3B).

As we expected, exposure to the bacteria strikingly induced the mRNA expression of all the four important pro-inflammatory cytokines/chemokines (*IL8*, *IL1A*, *IL6* and *CXCL2*) [17], [18] in the negative control cells. Intriguingly, knockdown of *ITGB5* and *MUC13* in IPEC-J2 cells both negatively regulated the expressions of *IL8*, *IL1A* and *CXCL2* mRNA in response to ETEC infection, and the decrease of *IL6* mRNA expression was also observed in *MUC13*-deficient cells (Figure 3).

In addition to the pro-inflammatory cytokines, in the F4ac ETEC infected negative control cells, there were significantly higher mRNA levels of *SLPI* (anti-inflammatory mediator) [19] and *PLAU* (encodes a serine protease which can in turn activate plasminogen into plasmin, and the latter is involved in degradation of the extracellular matrix) [20] than in the non-infected negative control cells (Figure 3). Highlighting the significance of *ITGB5* and *MUC13*, after exposure to ETEC *ITGB5*−/*MUC13*-deficient cells produced no more *SLPI* and *PLAU* than the non-infected negative control cells.

These results primarily revealed the pro-inflammatory activity of *MUC13* and *ITGB5* in porcine intestinal epithelial cells.

### *ITGB5* Depletion and *MUC13* Depletion Similarly Enhanced F4ac ETEC Adhesion to the IPEC-J2 Cells

**Figure 4:**
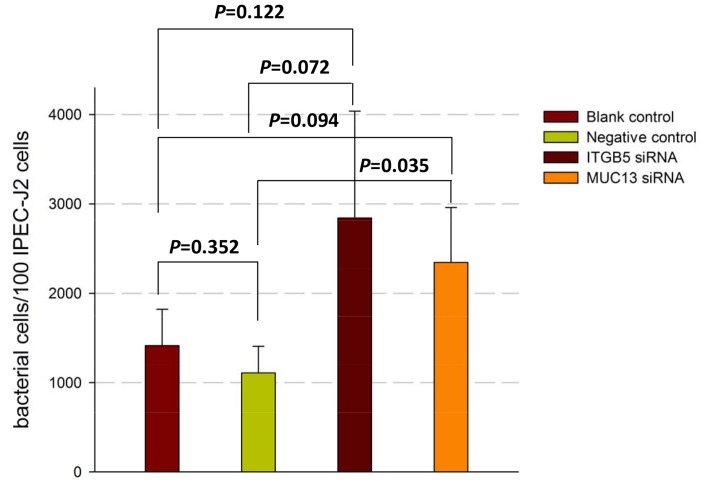
Overview of relative adhesion of F4ac ETEC to IPEC-J2 cells transfected with *ITGB5*-targeting/*MUC13*-targeting siRNA. IPEC-J2 cells were transfected with *ITGB5*-targeting and *MUC13*-targeting individually or negative control siRNA (Negative control) for 48 h and co-cultured with F4ac ETEC for 3 h. Adhesion of F4ac ETEC to the four group (including three transfected groups and one non-transfected group (Blank control)) cells were measured by real-time PCR. Statistics: mean ± SE; n = 3.

As both *ITGB5* and *MUC13* are candidate genes for ETEC F4ac receptor [3], [11], we finally assessed the influences of these two genes on adhesion of F4ac ETEC to the piglet small intestinal epithelial cells (Figure 4). IPEC-J2 cells were separately transfected with *ITGB5*- or *MUC13*-targeting siRNA for 48 h, and the cells were co-cultured with live bacteria for a further 3 h. Quantitative real-time PCR was used to measure the counts of adherent bacteria as described previously [5].

We observed that, compared with the negative control (transfected with control siRNA), knockdown of *ITGB5* and *MUC13* both increased the number of F4ac ETEC adhering to the porcine intestinal epithelial cells at the *P* value of 0.072 and 0.035, respectively. The enhancement of F4ac ETEC adhesion by deficiency of *ITGB5* or *MUC13* in IPEC-J2 cells is a novel observation.

## Discussion

In the present study, we primarily identified the effects of deficiency of *MUC13* or *ITGB5* in porcine intestinal cells upon F4ac ETEC infection and their mucosal immunogenicity. In human studies, gastric *MUC13* is found to be down-regulated after *H. pylori* infection [21] and has been shown to be aberrantly expressed in gastrointestinal cancers [22]. *ITGB5* is up-regulated in pancreatic adenocarcinoma [14],[23], and its repression impairs angiogenesis both *in vitro* and *in vivo* [24]. We have previously found that the expression of porcine *MUC13* and *MUC4* was down-regulated in IPEC-J2 cells post infection with the same F4ac ETEC strain, and the expression of *ITGB5* was not significantly decreased [5]. Indeed, our present study provided evidence for the first time that decreased endogenous *ITGB5* mRNA levels were associated with decreased mRNA levels of *MUC13* (Figure 1B), *MUC4*, *MUC20* (Figure 2A), *CXCL2*, *SLPI* and *PLAU* (Figure 3A), while decreased endogenous *MUC13* mRNA levels was associated with decreased *ITGB5* (Figure 1A), *MUC20* (Figure 2B), *IL8*, *IL1A*, *CXCL2* and *PLAU* mRNA (Figure 3B), in the *in vitro* porcine intestinal epithelial cells.

Up to now, the relationship between *MUC13* and *ITGB5* in swine is still undefined. Based on our study results, the deficiency of *MUC13* may suppress the expression of *ITGB5* (Figure S1). On the other hand, ITGB5 might regulate the expression of mucin genes (including *MUC13*), since Bianchi-Smiraglia *et al* found that integrin β5 deficiency induced both inhibitions of the Src-FAK and MEK-ERK signaling pathways [25], as the latter is essential for the activation of mucin genes transcription factor CREB (cAMP response element-binding protein) [26], . Moreover, in the current study we observed that silence of *ITGB5* decreased the expression of *MUC13* (Fold-change = −1.71, *P*<0.005; Figure 1B) while there was a small decrease in the expression of *ITGB5* in *MUC13*-KD cells (Fold-change = −1.34, *P*<0.01; Figure 1B). In our previous study [5], when the expression of *MUC13* was intensively reduced by F4ac ETEC infection, the mRNA expression level of *ITGB5* did not significantly decrease. The exact relationship of *ITGB5* and *MUC13* should be further tested by applying gene transfection of *ITGB5/MUC13* into IPEC-J2 cells or gene knockout of *ITGB5/MUC13* to confirm their effects on each other’s expression.

The relationships between *MUC13* and other paralogs such as *MUC4* and *MUC20* in porcine are unknown too, but no CNV (copy number variation) was detected in this region of pig chromosome 13 based on our Porcine SNP60 genotyping data [29] and copy number assay by quantitative real time PCR (our unpublished data). Studies have found strong correlation (be consistently up- or down-regulated) between the expression levels of genes that are located close to each other on the genome in mammals [16], [30]. Thus, the low co-expression of these genes could also partially and potentially be explained by *ITGB5*-targeting or *MUC13*-targeting RNA interference.

Our current study provides the first in vitro evidence that porcine *ITGB5* and *MUC13* have similar functions in modulating intestinal epithelial resistance to ETEC-induced inflammation. In human, Sheng *et al* [31] have proved that *MUC13* has modulation effect on chemokine (IL8 and CXCL2) secretion in LS513 intestinal epithelial cells through a nuclear factor-κB-dependent pathway. For human ITGB5, the pathways it is involved in include IL-8 signaling (INGENUITY TARGET EXPLORER), phagosome (conserved biosystem, from KEGG), immune system (organism-specific biosystem, from REACTOME) and antigen processing-cross presentation (organism-specific biosystem, from REACTOME). Published reports have shown that monocytes from three patients lacking of integrin β3 (*ITGB3*) expression were unable to produce interleukin 8 in response to *BLP* (SK4) [32]. Additionally, human *ITGB5* over-expression promotes the angiogenic properties of circulating angiogenic cells *via* Srckinase-mediated activation of *STAT3*, followed by increased transcription of *CXCL8* and *CCL2* [33]. Similar to the effect of human *ITGB3* and *ITGB5*, our data showed that the genetic ablation of *ITGB5* impaired responses of porcine intestinal epithelial cells to ETEC. A pathway including porcine *MUC13* and *ITGB5* has still not been defined. Based on the almost identical effect of the independent knockdown of the two adjacent genes in the present study, it implied that porcine *MUC13* and *ITGB5* may be involved in the same pathway which includes *IL8*, *CXCL2* and other immune related genes.

To date, only three well characterized porcine intestinal epithelial cell lines are available: IPEC-1, IPEC-J2 and IPI-2I [34]. Three major points demonstrate that IPEC-J2 represents a better model for this study than the two other cell lines: (I) Non-immortalized cell line IPEC-J2 can serve as a better model of the normal host intestinal epithelium than the transformed IPI-2I cell line [34], (II) Pavlova *et al.* showed that six F4 ETEC strains all failed to enhance IL-8 and TNFα mRNA expression in the IPI-2I cell line [35], and (III) porcine ileum is a site of minor importance for F4+ ETEC pathogenesis [36]. Furthermore, There are some RNAi studies performed in one cell line [37], [38], [39]. In order to prevent the time-caused conflict to harvest the cells for RNA extraction and for adhesion assay, respectively, we set the time point of harvesting cells (infected or non- infected with F4ac ETEC) for adhesion assay 4 h later than that harvesting cells (infected or non- infected with F4ac ETEC) for RNA extraction.

Adhesion of the F4ac ETEC to IPEC-J2 cells has been reported [5], [40]. Since *ITGB5* and *MUC13* are striking candidate genes for receptor of F4ac ETEC [7], [9], [11], the effects of them on ETEC-IPEC-J2 adhesion were firstly assessed. Surprisingly, knockdown of *ITGB5* and *MUC13* in IPEC-J2 cells both increased F4ac ETEC adhesion (Figure 4). Integrins, a structurally elaborate family of adhesion molecules, was reported to participate in a wide range of biological processes, including tissue repair, angiogenesis and inflammation [41]. The observation about *ITGB5* was in line with a published study showing that the absence of ITGB5 in Hela cells increased *Salmonella* invasion [42]. On the basis of published reports that bacterial pathogens often induce host cell apoptosis to promote their survival and dissemination [1], [43], [44], we speculated that the potential reason for increase of F4ac ETEC adhesion is that *ITGB5-*or *MUC13*-deficiency induced cell apoptosis and impair metabolic activity of IPEC-J2 cells.

In summary, our work demonstrated that the deficiency of endogenous *ITGB5* and *MUC13* can: (I) influence mRNA expression mutually; (II) affect the RNA levels of *MUC20*, *CXCL2* and *PLAU etc*; (III) inhibit the normal responses of intestinal epithelial cells to ETEC infection; (IV) promote F4ac ETEC to adhere to porcine intestinal epithelial cells *in vitro*.

## Materials and Methods

### Cell Culture and Bacterial Strain

Porcine intestinal cells (IPEC-J2) were grown in Dulbecco’s modified eagle medium (DMEM)/Ham’s F-12 (1∶1) medium (DMEM/F12, GIBCO, Invitrogen, Beijing) containing 5% fetal bovine serum, and were maintained in a 95% air-5% CO2 humidified atmosphere at 37°C and subcultured at 4-day intervals. F4ac ETEC strain 200 (O149:K91:F4ac, LT^+^, STa^+^, STb^+^, EAST1^+^) were removed from cryo-storage and cultured in Ordinary Broth Agar at 37°C for three generations (24 h per generation) [5].

### SiRNA Transfection

Small interfering RNAs (siRNAs) specifically targeting *MUC13* and *ITGB5* and the corresponding negative control (Stealth RNAi Negative Control Low GC Duplex) were chemically synthesized by Invitrogen (Carlsbad, CA). The sequences of small interfering RNAs were shown in Table 1. Porcine intestinal cells (IPEC-J2 cells) were transfected with siRNAs using Lipofectamine® RNAiMAX Transfection Reagent and Reverse Transfection protocol (Invitrogen) according to the manufacturer’s protocol. Briefly, after digesting IPEC-J2 cells in the logarithmic phase, cell suspension was prepared with a concentration of 100 cells/µl in complete growth medium without antibiotics. Diluted 3 µl gene-specific siRNA oligomers (20 µM) in 500 µl Opti-MEM®I reduced serum medium (Opti-MEM, Invitrogen) and added 5µl Lipofectamine® RNAiMAX to each well containing the diluted RNAi molecules. After 20 min incubation at room temperature, the complexes were added to one cell of the 6-well plate. To each well with RNAi duplex-Lipofectamine® RNAiMAX complexes, added 2500 µl of the diluted cell suspension. The IPEC-J2 cells incubated with transfection medium (500 µl Opti-MEM®I reduced serum medium +2500 µl complete growth medium) only were used as Blank control and that transfected with a non-target control siRNA were used as Negative control. Each transfection repeated three times.

### Infection of the Cell Lines

For enterotoxigenic *Escherichia coli* infection post siRNA transfection:

- At 44 hours post-transfection, monolayers of the cells in 6-well tissue culture plates were washed twice with PBS, and 1 ml of DMEM/F12 was added. A total of 20 µl of bacterial suspension of F4ac ETEC strain 200 (10^8^ CFU/ml, MOI (multiplicity of infection) = 8∶1) was added. Then, the cells and bacteria were co-incubated at 37°C in a 5% CO2- 95% air atmosphere for a further 3 h. These samples were used to RNA isolation.
- At 48 hours post-transfection, monolayers of cells in 6-well tissue culture plates were washed twice with PBS, and 1 ml of DMEM/F12 was added. A total of 30 µl of bacterial suspension of F4ac ETEC strain 200 (10^8^ CFU/ml, MOI = 10∶1) was added. Then, the cells and bacteria were co-incubated at 37°C in a 5% CO2-95% air atmosphere for a further 3 h. These samples were used to measure the adhesion values of the ETEC.

### RNA Preparation and Real-time RT-PCR

The IPEC-J2 cells used to isolate RNA were washed twice (*E.coli* non-infected) or four times (*E.coli* infected) with PBS, then lysed with TRIZOL Reagent (Life technologies, Carlsbad, CA, USA) directly in the culture dishes. Isolation of RNA was performed using TRIZOL Reagent following the manufacturer’s instructions.

According to the manufacturer’s instructions, complementary DNA (cDNA) was synthesized from the RNA using the Prime Script® RT reagent Kit with gDNA Eraser (Perfect Real Time) (TAKARA BIO, Dalian, China). The real time RT-PCR reactions were performed in a final volume of 20 µl with the Roche SYBR Green PCR Kit (Roche, Hercules, CA, USA) using a LightCycler® 480 Real-Time PCR System (Roche, Hercules, CA, USA). The relative RNA expression levels of siRNA targeting genes (*ITGB5*, *MUC13*), mucin genes (*MUC4*, *MUC20*), pro-inflammatory genes (*IL8*, *IL1A*, *IL6*, *CXCL2*), anti-inflammatory mediator *SLPI*, and *PLAU* were analyzed against housekeeping gene *ACTB* using the 2^−ΔΔCt^ (the gene primers were listed in Table 2). Duplicate qRT-PCRs were conducted on each sample and the average Ct was used for the analysis.

**Table 2:** Primers for quantitative real time PCR and RT-PCR.

Genes	GenBank Accession Number	Primers	Sequence
*MUC13* [5]	NM_001105293	F	*5′- GAGACTGGCTTTAGCAACCT-3′*
		R	*5′- AGTCTATCAAACCCTCACAC-3′*
*ITGB5* [5]	DQ786571	F	*5′-GACTGTCTGCTTATCCACCC-3′*
		R	*5′-CCATTCTTGACCAGGTTTGT-3′*
*MUC4* [5]	NM_001206344	F	*5′- AGGATGCCCAATGGCTCTACT-3′*
		R	*5′- AAGGAGGCTGGTTCCGTTGAT-3′*
*MUC20* [5]	NM_001113440	F	*5′- CAGCAAAGACCTCTAAGATGG-3′*
		R	*5′- CAGCAGGGAGACTTGGATGG-3′*
*IL8* [5]	NM_213867	F	*5′-CAAGCAAAAACCCATTCTCCG-3′*
		R	*5′-CCAGCACAGGAATGAGGCATA-3′*
*IL1A*	NM_214029	F	*5′-TAAGAATCTCAGAAACCCGAC-3′*
		R	*5′-GGCTGATTTGAAGTAGTCCAT-3′*
*CXCL2* [5]	NM_001001861	F	*5′- TGCAGACCGTGCAAGGAATT-3′*
		R	*5′- TGGCTATGACTTCCGTTTGGT-3′*
*IL6*	NM_214399	F	*5′-GAGAGCAATAAGGGAAATGTC-3′*
		R	*5′-TCTTCATCCACTCGTTCTGT-3′*
*SLPI*	NM_213870	F	*5′- CTGGGTGACTTAAAATGCTG-3′*
		R	*5′- CAAAGTAGATGGTGGTGGTA-3′*
*PLAU*	NM_213945	F	*5′-AAACCCTTCACTCCAGCACT-3′*
		R	*5′-TTGTCGGTACGGATCTTCAG-3′*
*ACTB* [5]	AY550069	F	*5′- GCTCTTCCAGCCCTCCTTCC-3′*
		R	*5′- ACAGCACCGTGTTGGCGTAG-3′*

[5] Reference No. 5.

### Adhesion Assay

The adhesion values of the F4ac ETEC strain 200 to IPEC-J2 cells were evaluated using a real-time quantitative PCR assay of *STa* gene as described previously [5]. As internal standards in this study we amplified serial dilutions of the respective bacteria in PBS ranging from 1×10^5^ to 1×10^2^ CFU/µl.

## Supporting Information

Figure S1 **Infection with F4ac ETEC did not influence the expression of** ***MUC13*** **or** ***ITGB5*** **in** ***MUC13*** **-KD and** ***ITGB5*** **-KD IPEC-J2 cells.**
(TIF)
