# Supplementary figures and images for: Gene Silencing of Porcine MUC13 and ITGB5: Candidate Genes towards Escherichia coli F4ac Adhesion
Source: PLoS One. 2013 Jul 29;8(7):e70303. doi: 10.1371/journal.pone.0070303 (PMC3726385; doi:10.1371/journal.pone.0070303)

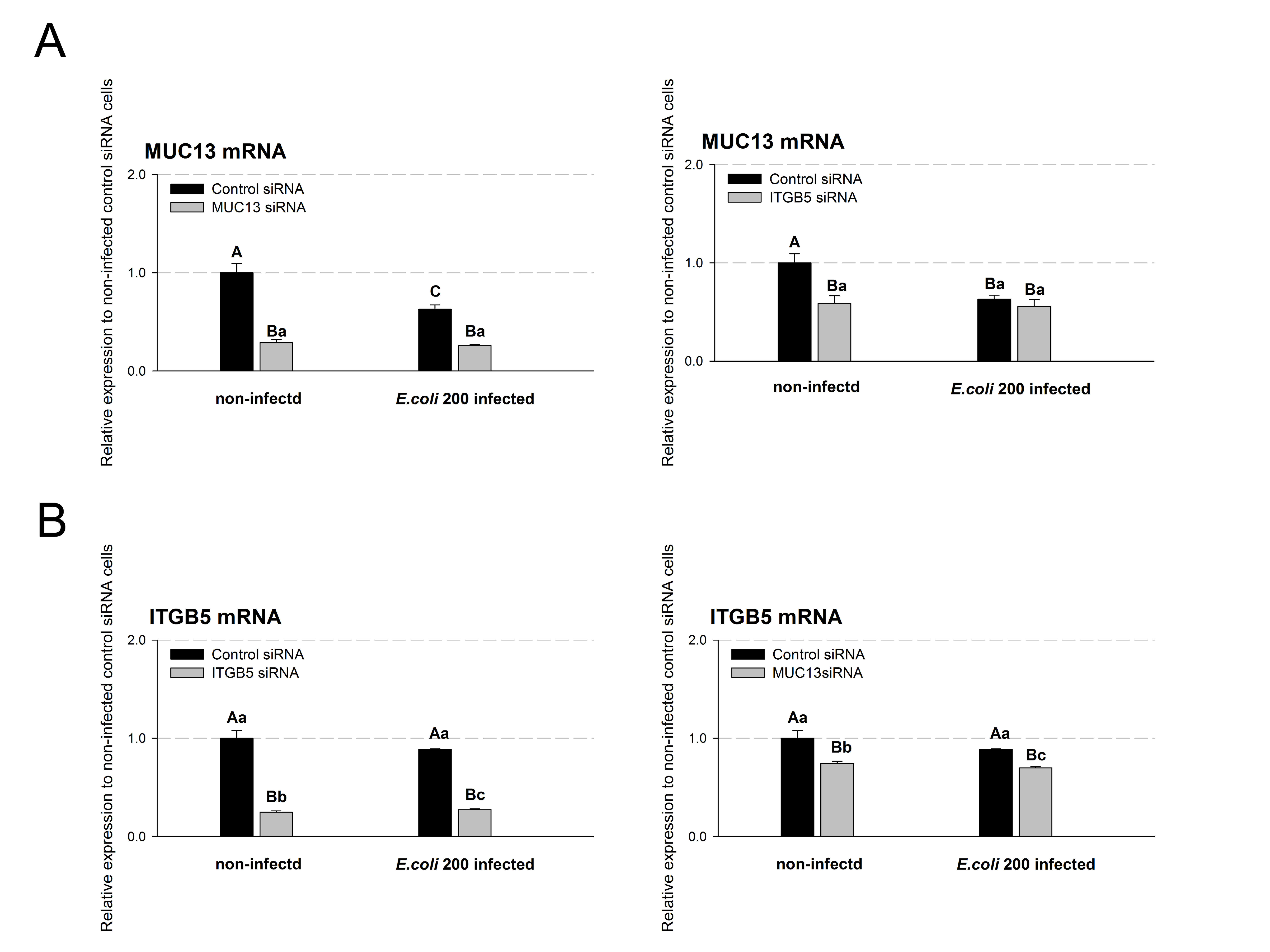

Supplement: Figure S1 — Infection with F4ac ETEC did not influence the expression of MUC13 or ITGB5 in MUC13 -KD and ITGB5 -KD IPEC-J2 cells. (TIF) [file pone.0070303.s001.tif]
